# Effects on and consequences of responses to errors: Results from two experimental studies

**DOI:** 10.1111/bjep.12686

**Published:** 2024-05-08

**Authors:** Maria Tulis, Markus Dresel

**Affiliations:** ^1^ University of Salzburg Salzburg Austria; ^2^ University of Augsburg Augsburg Germany

**Keywords:** adaptive reactions, beliefs, errors, learning, motivation

## Abstract

**Background:**

Interest in the potential of learning from errors to benefit innovation and organizational and personal growth is currently increasing. In practice, individuals frequently do not appear to learn spontaneously from errors and setbacks without support. Based on prior work, this paper considers antecedents and consequences of adaptive responses to errors.

**Aims:**

Two experiments with undergraduate students aimed to identify the causal link between beliefs and maintained motivation and the adaptation of actions to the end of analysing and effectively correcting errors.

**Samples and Methods:**

In Study 1 (*N* = 195, 72% female, *M* = 20.7 years, SD = 3.0), we experimentally manipulated learners' beliefs around the importance of errors to learning, after which they completed a 50‐min learning session on research methods and statistics. In Study 2 (*N* = 67, 58% female, *M* = 21.8 years, SD = 3.99), we intertwined the manipulation more closely with the actual learning process by using prompts about adaptive responses to errors immediately after error feedback.

**Results:**

In Study 1, those to whom we stressed the negative effects of errors showed significantly fewer adaptive action‐related responses to errors, less persistence, and less use of metacognitive strategies after errors. In Study 2, we found significant positive effects on the learners' persistence, their metacognitive control, and effort investment.

**Conclusions:**

Our results support and expand previous, mostly correlational, research findings on individuals' adaptive responses to errors.

## INTRODUCTION

### Errors and learning

Educationalists around the world would agree that errors – inattentive lapses aside – are sources of valuable information about a learner's potential misunderstandings, misconceptions, or gaps in their knowledge. Indeed, within the last decades, several studies, indicating differential effects of considering one's own errors (e.g., Metcalfe & Xu, 2018; Zhang & Fiorella, 2023), others' errors (e.g., Klopp & Stark, 2020) or worked examples of typical errors (e.g., Große & Renkl, 2007), have shown that errors can be effective at boosting learning by activating cognitive and metacognitive processes that are useful in eliciting self‐explanations, revising existing knowledge, and triggering conceptual change (e.g., Durkin & Rittle‐Johnson, 2012; Glogger‐Frey et al., 2015; Horvath et al., 2021; Kapur, 2013; Metcalfe et al., 2009; Safadi & Saadi, 2021). Theories have sought to explain learning from errors and failures from a largely cognitive perspective (Metcalfe, 2017; Tawfik et al., 2015, for an overview), relying primarily on processes of self‐explanation (Chi, 2000). Recently, Zhang and Fiorella (2023) provided a synthesis of the various theories. Alongside these, some researchers have attempted to attain a more comprehensive picture of learning from errors by including affective and motivational processes and characteristics of the learning environment in their analyses (e.g., Tulis et al., 2016). Their rationale for doing so takes account of a key difficulty that presents itself in relation to errors. Making errors is an unpleasant experience on the one hand, but necessarily an integral part of learning on the other hand, because tasks of a difficulty slightly above the learner's current skill level have the greatest potential for generating gains in learning and deeper understanding of a subject or competency (see, for example, Clifford, 1990).

### Errors and motivation

Due precisely to the aspect of challenge described above, however, errors will often go hand in hand with negative feelings and reduced motivation (Tulis & Dresel, 2018). While making an error may induce cognitive processing at a deeper level, it will simultaneously involve a high degree of emotional self‐reference, which may block the learner from engaging in a thorough analysis of the error and its causes (Horvath et al., 2021). More specifically, learners usually perceive errors as unpleasant, and thus potentially resulting in avoidant learning behaviour instead of increased effort investment and persistence to identify the underlying misconception (Eskreis‐Winkler & Fishbach, 2019). Such maladaptive reactions limit the use of the potential of errors to advance learning. It is therefore imperative for educators to pay attention to learners' engagement in the appropriate processes of self‐regulation after they make errors and receive feedback on them (Zamora et al., 2018). Learners may experience feedback on their errors as a threat to their self‐worth and find it undermines their motivation to achieve. In this context, we can consider the process of learning from errors to be a “specific learning phenomenon” (Tulis et al., 2016, p. 13); rather than simply “learning how to avoid specific errors in the future” (pointed out by Frese & Keith, 2015, p. 671). Instead, educators should support learners to emerge from this process with both improved “negative knowledge” on the subject at hand (Oser, 2018) and to develop a mindset that enables them to handle errors adaptively.

The bigger part of research on self‐regulation has focused on cognitive and metacognitive processes (Devolder et al., 2012), while the smaller part has addressed on emotional and motivational regulation (e.g., Daumiller & Dresel, 2019), specifically when it comes to exploring error situations (e.g., Reindl et al., 2020). Furthermore, experimental studies on the interplay of adaptive responses to mistakes are scarce (for an exception see Keith & Frese, 2005). The present study sought to fill this gap by systematically analysing the causality of error‐specific self‐regulatory processes, in terms of positive beliefs, affective‐motivational reactions and action‐related responses to errors, by means of two experiments.

### Why learning from errors requires adaptive reactions and responses

In line with Zhao (2011), we define learning from errors as an activity requiring effort. Errors occur when the result of an action deviates from an expected outcome or desired goal in an unintended manner (e.g., Hofmann & Frese, 2011); they serve in this way as an indicator of a need for something to change if the actor—in our context, the learner—is to achieve their aim. For instance, knowledge‐ and rule‐based mistakes stemming from an erroneous concept may signal a need for conceptual change (Tulis et al., 2016). We regard a learner's attempts, triggered by feedback on errors, to reduce such an unintended discrepancy as the starting point for learning from errors. However, learners differ in how they self‐regulate once aware of having made an error. The Dual Processing Model of Self‐regulation (Boekaerts & Niemivirta, 2000) provides an explanation for such differences on the basis of the threat to self‐worth and feared loss of resources the learner experiences after making an error. Learners who are primarily concerned with the negative consequences of errors first have to make use of adaptive emotional and motivational regulation strategies (Boekaerts, 2006) before they can focus on the error's causes, rethink their misconceptions and make corrections. Learners who focus on the potential of their errors for learning and personal growth may be more likely to concentrate on overcoming the misconception underlying their error and regulating their motivation to do this. In this context, *adaptive affective‐motivational reactions to errors* comprise the maintenance of learning motivation and functional emotions such as joy (Dresel et al., 2013; Tulis et al., 2011, 2016)—that is, they reflect the way in which learners self‐regulate their motivation to learn and the affect, specifically the negative deactivating affect, that is potentially associated with the detection of or feedback on errors. Alongside, *adaptive action‐related responses to errors* comprise learners' initiation of cognitive, particularly metacognitive, processes and behaviours with the specific aim of identifying and overcoming the possible misconception underlying the error in question. It is important to note here that a continuous dynamic links emotions, motivation, and their regulation, alongside cognitive processes and metacognitive activities, which means that all these entities and phenomena affect one another (Efklides et al., 2018). If learners are able to adapt positively in all these respects, they are more likely to commit to, and invest effort in, learning from their errors (Steuer et al., 2013). In view of these insights from previous work, we expected positive associations between both types of adaptive reactions to error feedback, and persistence in the face of challenge and setbacks, in order to gain a deeper understanding of the causes underlying the error and how to correct it (Metcalfe, 2017). Furthermore, we investigated the positive effects of beliefs about errors as learning opportunities.

### Beliefs that errors can constitute learning opportunities

Positive error‐related beliefs (in the sense of values or opinions which may guide one's behaviour or actions; Buehl & Alexander, 2009) comprise the understanding that errors are useful and necessary ingredients of learning (Tulis et al., 2018). Positive error‐related beliefs may support students' self‐regulation and aid them in engaging with challenging learning situations, despite the risk of making errors in the process. Findings that indicate a positive correlation linking adaptive affective‐motivational reactions and action‐related responses to errors to beliefs that errors can represent learning opportunities (Tulis et al., 2018) are in agreement with this assumption. Support also comes from attitude research which basically notes that specific beliefs may foster engagement in specific behaviours in which the issue of the belief is prevalent (Ajzen, 2012). These indications from previous work lead us to expect, in theoretical terms, that positive error‐related beliefs may serve as a guidance and may therefore lead to adaptive affective‐motivational reactions and action‐related responses of learners after receiving feedback on errors. We note further evidence that may undergird this assumption in findings that learners' attitudes towards errors predict their learning behaviour in various situations (Leighton et al., 2018).

Existing research on learners' beliefs about errors is sparse (one example is Tulis et al., 2018). The same is the case for work on learners' attitudes to errors, in terms of their thoughts and opinions around the role of errors in learning (attitudinal cognition), emotions associated with errors (attitudinal affect), and the tendency to behave in particular ways when confronted with errors (attitudinal behaviour). One instance of research addressing these matters is the study by Leighton et al. (2018), which sets out a generic instrument for ascertaining university students' beliefs about errors. It has found that students' attitudinal behaviours around errors predicted academic achievement and were indirectly influenced by attitudinal affect regarding errors, which in turn showed a positive association with students' attitudinal cognitions (i.e., beliefs) about errors. Already in the late 1990s, Rybowiak et al. (1999) had developed a questionnaire and proposed several components of error‐specific attitudes that incorporated a positive view of the beneficial role of errors in individual improvement – that is, a view of errors as opportunities for learning – alongside “error risk‐taking” and a general flexibility and openness towards errors. Tulis et al. (2018) used a five‐item scale to measure error‐related beliefs to the end of providing empirical evidence of these beliefs' capacity to predict students' affective‐motivational reactions and action‐related responses to errors, above and beyond self‐concepts and goal orientations. Alongside findings from correlational work, intervention studies on effective error management emphasize the significance of error‐related beliefs. The studies conducted by Keith (including Keith & Frese, 2005), in which the researchers provided prompts on the positive utility of errors while participants practised a task, can serve as an example in this regard. Prompts direct learners' attention to important aspects of an activity during the learning process (Quintana et al., 2004). Keith and Frese (2005) found medium to large effects on transfer test performance in the treatment condition, as compared to error‐avoidant training, with emotional self‐regulation and metacognitive activities mediating this effectiveness. Similarly, Cillarege et al. (2003) found that error management training for mature learners (aged 40 years and above), including reflection on and questioning of their beliefs around errors, led to higher levels of positive affect after making errors and to higher performance test scores.

### Desiderata for research

Notwithstanding an increasing tendency to celebrate errors as “teachable moments” and rising interest in the importance of risking errors if innovation and growth are to occur (see, for example, Maxwell, 2000), not all learners may hold positive beliefs around the potential of errors to promote personal growth. Instead, cumulative experiences with academic “failure” and negative implications of errors made in challenging situations where performance is critical may well inform negative beliefs surrounding errors, beliefs which preclude their recognition as learning opportunities (see, for instance, Mason & Singh, 2010). Beyond the studies cited above, however, we still know little about the causal effects of learners' beliefs around errors, a state of affairs underlined by the lack of experimental research in this field identified in a recent meta‐analysis of experimental studies on learning from failure (Darabi et al., 2018).

In addition, when supporting and guiding learners, educators need to consider the complex interplay of cognitive, metacognitive, emotional, and motivational aspects of error‐making. In order to develop evidence‐based programs for educators, or to implement effective scaffolding within learning environments in the future, we need research findings on the causal interplay of those aspects. To date, research on individual responses to errors have been predominantly correlative – except some training/intervention studies in organizational contexts (Keith & Frese, 2008), or recent attempts to identify the causal impact of error‐related teaching strategies on primary school pupils' perceived error climate (Soncini et al., 2020). The present research aimed to help filling these gaps.

## RESEARCH QUESTIONS AND OVERVIEW OF THE STUDIES

Our studies, drawing on the review of the literature and on previous research findings which point to the potential of supporting learners to benefit from their errors, pursued the principal purpose of investigating the causal interplay of positive beliefs and adaptive responses to errors in terms of motivational and metacognitive self‐regulation, and persistence in the face of feedback on errors in an authentic learning setting. More specifically, Study 1 focused on the alteration of error‐related beliefs via the provision of written “tips for learning” to students prior to the learning session that encompassed the completion of tasks and feedback on errors made, while Study 2 addressed both types of adaptivity (affective‐motivational reactions and action‐related responses) directly via prompts given immediately after error feedback within the digital learning environment. In those conditions, we expected that more positive beliefs about errors as learning opportunities would cause an increase in adaptive affective‐motivational reactions and adaptive action‐related responses to errors. Moreover, we expected that these two types of adaptivity to errors would, in turn, be causally related to increased metacognitive strategy use, effort, and persistence, as well as to larger knowledge gains.

More precisely, in *Study 1*, we assumed that the induction of positive beliefs about errors prior to the learning session would have positive effects, while the induction of negative beliefs centring error prevention would cause negative effects on variables conducive to learning from errors. Specifically, we tested the following hypotheses:

Learners in the experimental condition in which the positive role of errors in learning was emphasized (i.e., experimental group/EG 1: positive beliefs about errors) report the most adaptive responses to their errors. Learners who received “tips” with an emphasis on negative error‐related beliefs (i.e., experimental condition following an error prevention approach, EG 2) report the least adaptive responses. In detail, we expected the following significant differences in Study 1:

- the highest adaptive affective‐motivational reactions in EG 1, and lowest in EG 2;
- the highest adaptive action‐related responses in EG 1, and lowest in EG 2;
- the greatest use of metacognitive strategies in EG 1, and lowest in EG 2; and
- the highest persistence in the face of error feedback (i.e., repeating the task instead of continuing with the next section) in EG 1, lowest in EG 2.

*Study 2*, the second experiment used more direct prompts, placed immediately following feedback on errors, to the end of eliciting learners' adaptive affective‐motivational reactions and action‐related error responses (Dresel et al., 2013), and learners' effort investment, and persistence. More specifically, we tested the following hypotheses:

The provision of affective‐motivational prompts acts particularly to promote effects on students' investment of effort in continued learning despite obstacles and their persistence.

The provision of action‐related adaptivity prompts acts particularly to promote effects on students' use of metacognitive strategies (i.e., identifying the necessity of changing how to approach the task).

In both studies, we expected greater gains in knowledge (post‐test scores) in the positive belief condition (Study 1) and in the condition with a combination of affective‐motivational and action‐related adaptivity prompts (Study 2) than in the control group in each study.

## MATERIALS AND METHODS

**FIGURE 1:**
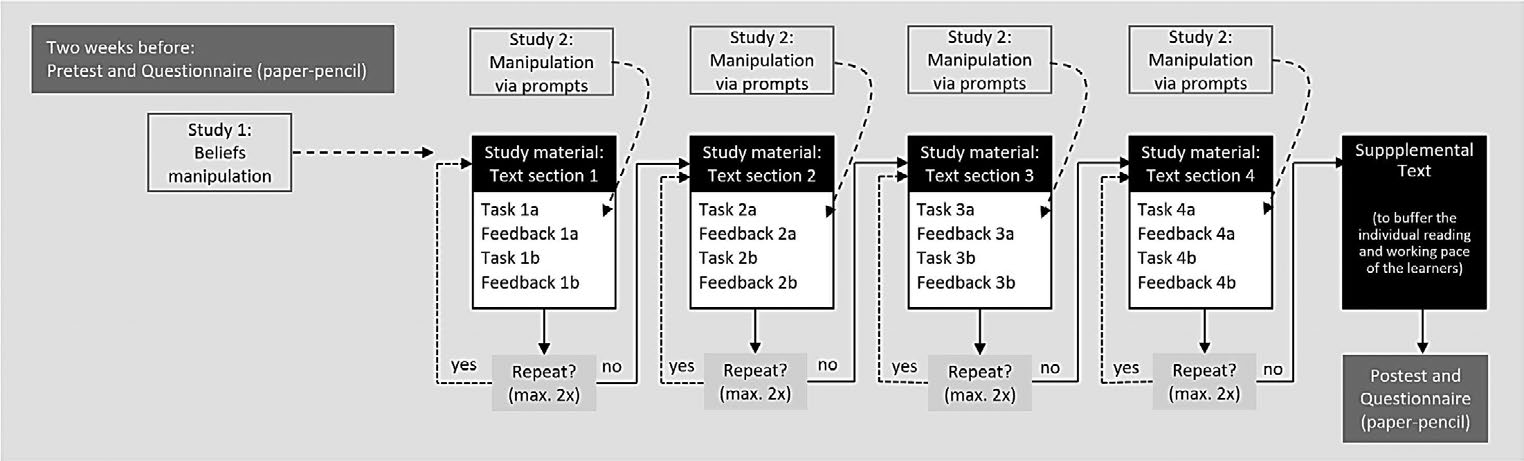
Computer‐based learning setting and experimental design for Study 1 (manipulation before the 50‐min learning session) and Study 2 (manipulation via prompts directly after error feedback in each learning section).

Participants in both studies were undergraduate students inscribed in teacher studies in a German university. Both studies took place in the computer lab, each participant working individually at a computer. Within a 50‐min learning session on research methods and statistics, participants worked through four sections of text with information they were intended to absorb and retain, and the corresponding practice tasks/quizzes, with immediate feedback and the opportunity to repeat each section if errors had occurred (Figure 1). The quizzes were designed in such a way that they were difficult but solvable by means of the learning content. We randomly assigned the participants in both studies to one of the experimental or control conditions, which we will describe separately for each study in the sections that follow. In each study, a manipulation check was conducted. We collected all data approximately two weeks before (T1: pre‐test of knowledge and baseline questionnaire) and directly after the 50‐min learning session (T2: post‐test of knowledge and questionnaire). In Study 1, we additionally assessed beliefs about errors after the manipulation (before the participants commenced the learning programme).

On completion, participants were given the knowledge post‐test and questionnaire and received a debrief on the experimental design and on the positive effects of errors on learning. The studies received University Ethics Committee approval, and all participants gave their informed consent to participation and inclusion.

### Measures: Dependent variables

All questionnaire items used Likert‐type scales ranging from 1 (*strongly disagree*) to 6 (*strongly agree*). The experiments generally focused on the relevant domain of study in T1 and on the specific learning environment in T2 (“When learning about research methods in this session …”).

#### Beliefs about errors

We assessed error‐related beliefs in Study 1 using a five‐item scale from Tulis et al. (2018). Internal consistency was satisfactory (*α* = .95). One sample item is: “I can learn something from my errors”. Participants in Study 1 completed the five‐item scale for error‐related beliefs not only after but also at the beginning of the learning session (i.e., after the manipulation).

#### Adaptive affective‐motivational reactions to errors

This scale from Dresel et al. (2013) comprised six items (*α* = .84–.88, Study 1 and Study 2), such as “When I made an error in the learning session, I still felt like continuing to work” (post‐questionnaire).

#### Adaptive action‐related responses to errors

This scale from Dresel et al. (2013) comprised seven items (*α* = .82–.87, Study 1 and Study 2), such as “When I did something wrong in the learning session, I specifically tried to work out what the problem was” (post‐questionnaire).

#### Metacognitive strategy use

Eight items, constructed by extending and adapting a five‐item scale by Gold and Souvignier (2004), assessed metacognitive control in the face of difficulties. One example of an item is: “If I found something difficult during learning in the session, I changed my approach” (*α* = .89–.81, Study 1 and Study 2).

We measured *persistence* by dividing all selected repeat options by all repeat options (a maximum of two options per section = 2 × 4 sections = 8).

#### Gains in knowledge/knowledge of research methods

We assessed prior knowledge via four multiple‐choice tasks, each with four options which functioned as items (see supplemental material, S1); the addition of four further tasks in the post‐test resulted in a total of eight multiple‐choice tasks in the post‐test at T2. The analysis covered the proportion of items answered correctly by the participants.

#### Investment of effort

In Study 2, we additionally assessed the self‐reported effort students invested in the learning environment (10 items, *α* = .95, an extended version of the subscale of Wild & Schiefele, 1994) at T2 (e.g., “I made an effort even when the task was difficult”).

### Study 1: Participants and procedure

Study 1 followed a between‐subjects design with three conditions and with beliefs about the function of errors for learning as an independent variable: EG 1 emphasized the benefits of errors, while EG 2 informed participants that errors interfere with the acquisition and retention of correct concepts, draw learners' attention to inaccurate facts, and have detrimental effects on effective learning. The CG condition did not draw a connection between errors and learning. To determine the sample size, we conducted an a‐priori power analysis using G*Power 3.1 (Erdfelder et al., 1996), which indicated a sample of 53 participants per group, with medium effect size, 80% of power and alpha error probability set to .05. The sample consisted of *N* = 195 teaching degree undergraduates from Germany (72% female, *M* = 20.7 years, SD = 3.0). Most of the participants (86.2%) were in their first year of their studies; 9.7 percent of participants were in their third semester (beginning of second year).

At the beginning of the computer‐based learning session, participants received a booklet with “tips”. Its content for the experimental groups was “tips for learning,”, while the control group (*n* = 63) was given “tips on action to take in case of fire”; this appeared as a suitable cover story, as a fire safety drill had taken place at the university some weeks previously. The “tips for learning” highlighted either the positive (EG 1; *n* = 60) or negative effects of errors (EG 2; *n* = 72). All three booklets were equal in length, structure and layout; an example booklet from EG 1 (in German language) is provided in the supplemental material (S2).

The manipulation in the booklet took place in three stages: First, readers received a piece of informational text, framed as personally relevant to them (as university students/learners) and as based on high‐quality research findings. Second, they saw a pair of role models (typical student figures, one male, one female) and read speech bubbles in which these figures spoke about situations in their degree courses and their thoughts on errors in learning (examples are provided in the supplemental material, S3). Third, we asked participants to write down, in their own words and in as much detail as possible, their ideas on the role of errors in learning. In addition to this elaborated reproduction of the content of the tips they have read, they were encouraged to write down one sentence (a “take‐home message”) on a post‐it note provided in the booklet and to stick it to their computer screen during the learning session (an example is provided in the supplemental material, S4). This message served to further reinforce the manipulation during the activity of learning.

#### Manipulation check

To check whether participants correctly perceived the manipulation—that is, positive (EG 1), and negative (EG 2) error‐related beliefs—two independent and blind raters categorized participants' written narratives on the content of the tips. The manipulation proved highly effective (Table 1A,B). One example for the written responses of the participants in EG 1 read:

“Errors help you to recognise that you are at the right level of difficulty, because if you don't make errors, the tasks are often too easy for you. By recognising your errors, you can specifically look for the cause and try to avoid the same error in the further learning process. Errors are useful for learning, as you can recognise where there are still gaps and what you should practise in more detail. They may give you a new perspective on the subject matter and help you understand connections that you didn't see before.” (JISL27/1/2)

**TABLE 1:** Manipulation check (Study 1): Elaborative writing task.

A. Categories (experimental group 1)	*f*	%
Errors are normal/natural/human.	30	60.0
Errors show what still needs to be learned and help to close knowledge gaps.	26	52.0
Errors indicate that the tasks are within the ideal difficulty range.	25	50.0
Errors point out your own weaknesses and illustrate what you cannot do yet.	24	48.0
Errors provide a learning opportunity/learn something new/effective learning.	20	40.0
Errors are necessary because one cannot understand new content right away.	18	36.0
Errors promote a more intense exploration of the learning material.	18	36.0
Errors can improve one's own skills and help to attain learning gains.	19	20.0
Errors will not be repeated.	9	18.0
Errors are useful.	8	16.0
Learning is not possible without errors.	6	12.0
Other	18	36.0

B. Categories (experimental group 2)	*f*	%
Errors make you to memorize the wrong information instead of the right one.	29	69.0
Errors distract from your own skills/abilities.	28	66.7
Errors disrupt learning/interfere with learning.	24	57.1
Errors cause confusion and uncertainty.	20	47.6
Errors demotivate/negatively affect self‐confidence/evoke negative emotions.	19	45.2
Errors indicate deficiencies and incompetence.	13	31.0
Errors result in a large expenditure of time/waste of time.	9	21.4
Errors have a negative effect on learning processes/outcomes/performance.	9	21.4
Errors slow down/impede learning processes.	8	19.0
Errors provide little learning potential.	7	16.7
Other	3	7.1

For EG 2, the following two examples from the participants' written narratives illustrate their negative view of errors as a result of the manipulation text:

“If you concentrate too much on your errors when studying, this leads to poorer performance in exams. You only practise the errors and not the right things. You shouldn't waste too much time revising your errors, as this consolidates incorrect knowledge. You can hardly learn anything from errors because at most they show you your deficits, they are demotivating, and they are not important for the exam. They disrupt your learning as you no longer concentrate on what you already know. Errors interfere with learning because they distract you from what is important and correct.” (BGDE13/3/3)

**FIGURE 2:**
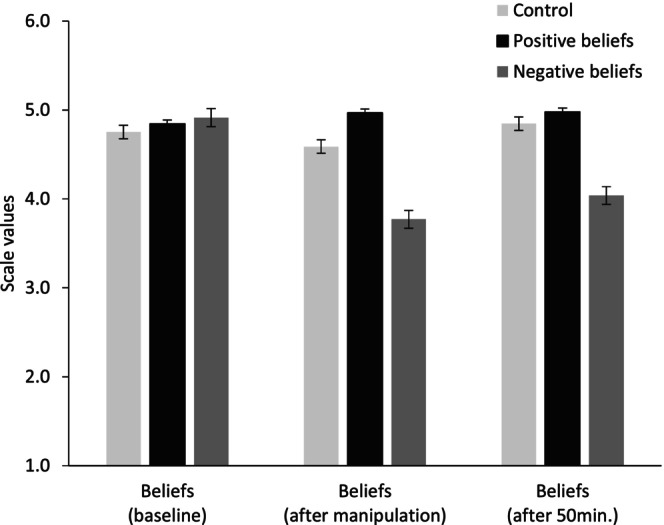
Beliefs about errors in Study 1 (mean scores for beliefs at baseline, directly after manipulation, and after the 50‐min learning session).

As a second manipulation check, we analysed the “take‐home message” participants had written on the post‐it notes. Those proved an effective manipulation as well (examples are presented in the supplemental material, S4). Finally, group comparisons demonstrated differences in participants' error‐related beliefs after the manipulation (*F*_(2,192)_ = 32.48, *p* < .001, η_p_^2^ = .25) in the expected directions: in EG 1, students reported the strongest positive beliefs about errors as learning opportunities, followed by the control group, and EG 2 with showing the lowest positive beliefs (see Results, Figure 2, error bars show standard errors of the mean).

### Study 2: Participants and procedure

Prompts direct learners' attention to important aspects of an activity during learning, and they may benefit learning either by addressing error identification and explanation (see, for example, Siegler, 2002) or by targeting motivational self‐regulation (e.g., Daumiller & Dresel, 2019). Study 2 followed a between‐subjects design with four conditions and with affective‐motivational prompts or action‐related prompts or a combination of both as an independent variable. Participants in Study 2 (*N* = 61, 58% female, *M* = 21.8 years, SD = 3.99, teaching degree undergraduates: first year of study: 51%; second year of study: 27.5%, third year of study: 10.7%, fourth year of study: 7.4%) were randomly assigned to one of four experimental conditions, receiving either no prompts (CG: *n* = 16), prompts inducing adaptive affective‐motivational reactions to errors (EG 1: *n* = 14), prompts inducing adaptive action‐related responses to errors (EG 2: *n* = 16), or both types of prompts (EG 3: *n* = 15). The prompts appeared in the digital learning environment during the learning session, immediately after error feedback. We therefore expected larger effects on the dependent variables.

#### Manipulation check

Participants' mean scores at T2 on the scales assessing affective‐motivational reactions and action‐related responses to errors were used as manipulation check. Group comparisons demonstrated significant differences in the expected directions for action‐related error reasponses (*F*_(3,60)_ = 3.20, *p* = .03, η_p_^2^ = .15): highest mean scores in EG 3, followed by EG 2, then EG 1, and lowest mean scores in the CG (see Results, Figure 4). However, no significant group differences were found for affective‐motivational reactions to errors.

## RESULTS

**FIGURE 3:**
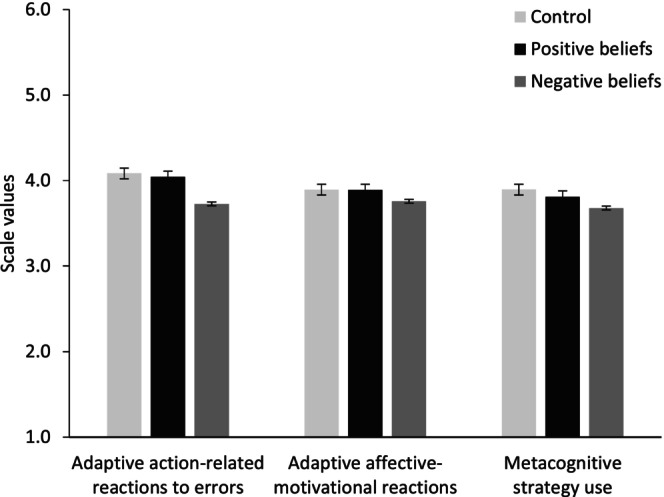
Group comparison on dependent variables (means scores at T2, after completing the 50‐min learning session) in Study 1.

Descriptive statistics and bivariate correlations for both studies can be found in Tables 2 and 3. In Study 1, as expected, we found no differences in error‐related beliefs at T1, and significant group differences directly after the manipulation: *F*_(2,192)_ = 32.48, *p* < .001, η_p_^2^ = .25 with medium to large effect sizes (EG 1 > CG: *d* = .63, CG > EG 2: *d* = .85). Figure 2 displays the ANOVA results on participants' beliefs about errors (error bars show standard errors of the mean). However, the effect of the induction of positive error‐related beliefs diminished after 50 min, whereas the induction of beliefs addressing negative effects of errors on learning remained stable over time. At the end of the learning session only the effect of the manipulation in EG 2 was still prevalent: *F*_(2,187)_ = 21.41, *p* < .001, η_p_^2^ = .19; CG > EG 2: *d* = .85. Regarding the other dependent variables, there was no statistically significant difference between CG and EG 1. There were significant group differences in action‐related responses to errors (*F*_(2,192)_ = 3.30, *p* = .04, η_p_^2^ = .033). Specifically, planned contrasts revealed significant differences between EG 1 (*M* = 4.04; SD = .97) and EG 2 (*M* = 3.73, SD = .89; contrast estimate ψ = .32, *p* = .04, *d* = .36) as well as between the CG (*M* = 4.08, SD = .78) and EG 2 (ψ = .36; *p* = .02, *d* = .41). Thus, participants in EG 2 showed significantly fewer action‐related responses to errors. Students in EG 2 were less persistent (see Table 4) and, compared to CG, reported lower metacognitive control (i.e., the use of metacognitive strategies in the face of difficulties: *t*(127) = 1.92, *p* = .04 (one‐tailed), *d* = .31 (Figure 3; error bars show standard errors of the mean). Regarding adaptive affective‐motivational reactions to errors no differences were prevalent. Thus, hypothesis H1 was partly supported, as learners in EG 2 reported the least adaptivity in terms of action‐related error responses (a), metacognitive activity (c), and persistence (d), but not for adaptive affective‐motivational reactions to errors (b). Furthermore, the expected positive effects in EG 1 were not evident in our data. Finally, no group differences were found with respect to knowledge gains from pre‐ to post‐test (H4).

**TABLE 2:** Bivariate correlations beliefs and adaptive responses to errors (Study 1 above the diagonal, Study 2 below the diagonal).

		2	3	4	5	6	7	8	9	10
1	Beliefs about errors (after manipulation, only Study 1)	.30**	.77**	.20**	.26**	.12	.27**	.23**	.13	.09
2	Beliefs about errors (T1)		.29**	.53**	.16*	.36**	.25**	.16*	.13	.03
3	Beliefs about errors (T2)	.27*		.28**	.37**	.15*	.28**	.32**	17*	.01
4	Adaptive action‐related error reactions (T1)	.45**	.16*		.28**	.26**	.20**	.27**	.03	−.03
5	Adaptive action‐related error reactions (T2)	.12	.45**°°	.20*		.00	.35**	.66**	.56**	.16*
6	Adaptive affective‐motivational error reactions (T1)	.30**	.12	.27*	.04		.37**	.00	.00	−.07
7	Adaptive affective‐motivational error reactions (T2)	.18*	.20*	.19*	.21*	.32**		.35**	.21**	.04
8	Metacognitive strategy use (T2)	.13	.42**	.23*	.70**	.05	.17*°		.40**	.19*
9	Persistence index	.09	.15	.09	.37**	.02	.23*°	.27*		.19*
10	Knowledge post‐test (T2)	.04	.14	−.01	.12	.19*	.06	.20*	.16*	
11	Effort investment (T2, only Study 2)	.12	.33**	.01	.58**	.01	.09°	.25*	.25*	.16*

*Note*: * *p* < .05 (2‐tailed). ** *p* < .01 (2‐tailed), Study 1: *N* = 195, Study 2: *N* = 67.

**TABLE 3:** Descriptive statistics for Study 1 and Study 2.

		Study 1		Study 2	
		*M*	SD	*M*	SD
1	Beliefs about errors (after manipulation, only Study 1)	4.40	1.05	‐	‐
2	Beliefs about errors (T1)	4.83	0.70	4.75	0.68
3	Beliefs about errors (T2)	4.60	1.04	4.90	0.77
4	Adaptive action‐related error reactions (T1)	4.55	0.60	4.62	0.75
5	Adaptive action‐related error reactions (T2)	3.94	0.90	4.51	0.78
6	Adaptive affective‐motivational error reactions (T1)	3.78	0.85	3.67	0.81
7	Adaptive affective‐motivational error reactions (T2)	3.84	0.77	4.19	0.93
8	Metacognitive strategy use (T2)	3.79	0.80	4.14	0.71
9	Persistence index	0.44	0.37	0.71	0.29
10	Knowledge post‐test (T2)	21.56	4.00	20.96	4.60
11	Effort investment (T2, only Study 2)	‐	‐	4.09	0.80

*Note*: Study 1: *N* = 195, Study 2: *N* = 67.

**TABLE 4:** Participants decisions to continue with the next or retry after error feedback (Study 1).

Retry	Total			EG 1				CG				EG 2			
	*n*	*f*	%	*n*	*Incorrect* (one/both)	*f*	%	*n*	*Incorrect* (one/both)	*f*	%	*n*	*Incorrect* (one/both)	*f*	%
Persistence11	195	94	47.2	60	(28/18)	31	51.7	63	(31/16)	33	52.4	72	(39/17)	30	41.7
Persistence12	94	12	14.3	31		4	13.0	33		5	15.1	30		3	10.0
Persistence21	195	59	29.6	60	(26/13)	20	33.3	63	(28/14)	24	38.1	72	(31/10)	15	20.8
Persistence22	59	16	27.1	20		5	25.0	24		7	29.2	15		4	26.7
Persistence31	195	91	45.7	60	(13/45)	34	56.7	63	(17/43)	32	50.8	72	(23/45)	25	34.7
Persistence32	91	44	48.3	34		18	53.0	32		18	56.2	25		8	32.0
Persistence41	195	77	38.7	60	(26/27)	24	40.0	63	(33/23)	26	41.3	72	(33/30)	27	37.5
Persistence42	77	30	39.0	24		9	37.5	26		9	34.6	27		12	44.4

*Note*: EG 1 = experimental group 1, EG 2 = experimental group 2, CG = control group, *n* = number of students, *f* indicates the absolute frequency of participants who opted for a retry. “Retry” comprises the categories “persistenceX1” (one retry) and “persistenceX2” (second retry), with X for the learning text section 1–4. Per section the maximum number of possible repetitions were 2 times; “*incorrect* (one/both)” represents in brackets the number of having one task incorrect/number of having both tasks incorrect (within‐subjects).

**FIGURE 4:**
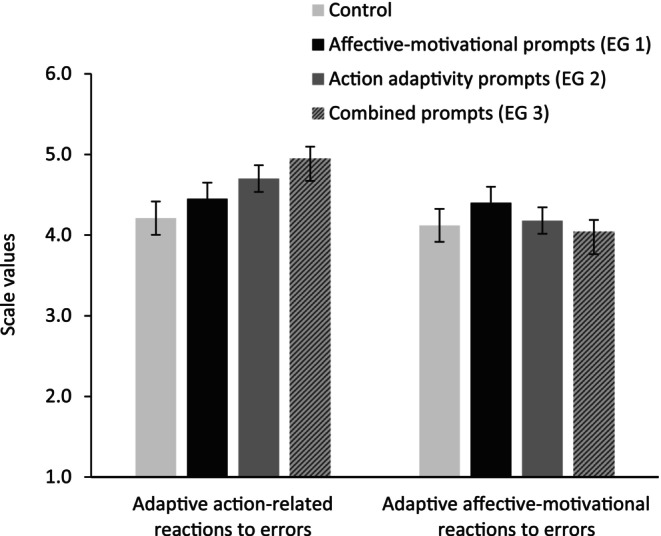
Mean scores for adaptive action‐related and affective‐motivational reactions to errors after the 50‐min learning session in Study 2, split by groups.

**FIGURE 5:**
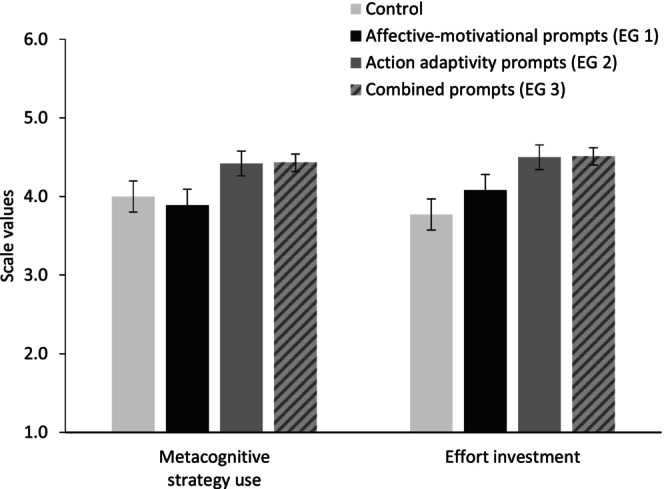
Mean scores for metacognitive strategy use and effort investment (T2) in Study 2, split by groups.

**FIGURE 6:**
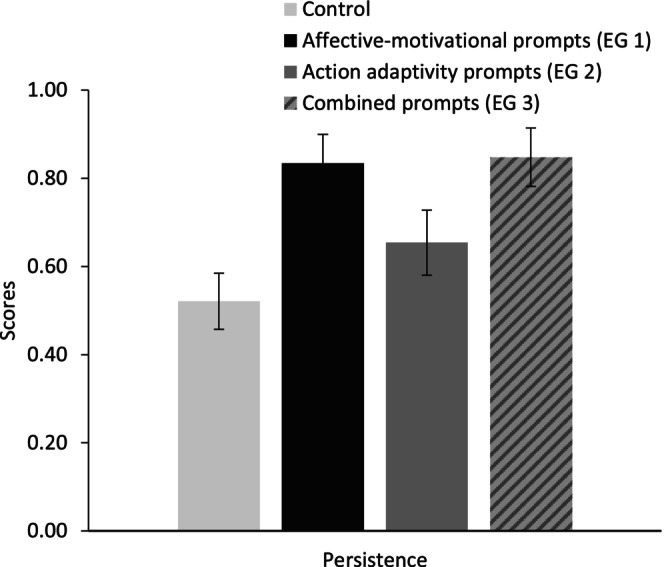
Mean scores for persistence in Study 2, split by groups.

Figure 4 displays group differences in participants' adaptive affective‐motivational reactions and action‐related error responses in Study 2. Concerning the dependent variables in Study 2, there were significant effects in metacognitive strategy use (*F*_(3,60)_ = 3.03, *p* = .04, η_p_^2^ = .14), effort investment (*F*_(3,60)_ = 3.33, *p* = .03, η_p_^2^ = .15), and persistence (*F*_(3,60)_ = 5.34, *p* < .01, η_p_^2^ = .22). Planned contrasts, comparing the experimental conditions (EG 1, EG 2, and EG 3) to the control group (CG) as well as the experimental groups with affective‐motivational prompts (EG 1 and EG 3) and action‐related prompts (EG 2 and EG 3) to each other and to the control group yielded significant differences. More specifically, EG 2 showed higher metacognitive scores compared to EG 1 (ψ = .53, *p* = .03, *d* = .77) and tended to show higher metacognitive strategy use compared to CG (ψ = .42, *p* = .06, *d* = .62). Metacognitive strategy use tended to be higher in EG 3 (action‐related prompts combined with affective‐motivational prompts) compared to CG (ψ = .44, *p* = .05; *d* = .73) and was higher than in EG 1 (ψ = .54, *p* = .02; *d* = .95). Thus, in line with the hypothesis (H3), participants in the action‐related prompts conditions reported a higher use of metacognitive strategies than those in the affective‐motivational prompts condition and the control group (see Figure 5). Thus, hypothesis H3 was supported. Contrary to our expectations (H_2_), effort investment was not highest in EG 1—but in the combined prompts condition (ψ = .74, *p* = .01; *d* = .86)—but also in the adaptive action‐related prompts condition (ψ = .73, *p* = .01; *d* = 1.01) compared to the control group (see Figure 5). However, in accordance with our expectations, persistence was significantly higher in the combined prompts condition (ψ = .33, *p* = .001; *d* = 1.27) and the adaptive affective‐motivational prompts condition (ψ = .30, *p* < .01; *d* = 1.15) compared to the control group (Figure 6, error bars show means and standard error of the mean). Again, no effects on post‐test knowledge were found.

## DISCUSSION

We conducted two experiments with undergraduate students that aimed to identify the causal link between positive beliefs and responses to errors, and how these influence learners in maintaining their motivation, and help learners adapt their actions to the end of analysing and effectively correcting their errors. In Study 1, we manipulated learners' error‐related beliefs prior to a learning session. In Study 2, we used specific prompts for adaptive responses to errors following error feedback. Our results support and expand previous, mostly correlational, research findings.

In Study 1, in EG 1, we emphasized the potential of errors for learning and personal growth; in EG 2, by contrast, we highlighted the potential harm errors can do to learning and to the recall of correct information (i.e., we emphasized the benefits of error prevention). Findings from Study 1 indicate that those to whom we stressed the deleterious effects of errors (EG 2) showed significantly fewer action‐related responses to errors, less persistence, and less use of metacognitive strategies. This said, we did not find a positive effect on responses to errors in EG 1, in which we manipulated beliefs on errors in a “positive” manner.

Our results suggest that while manipulation proved effective in the short term, more stable changes in positive error‐related beliefs and attitudes seem more difficult to achieve. In EG 1, we have pointed out the adaptive function of errors for learning in a very brief intervention, in the form of “learning tips” at the beginning of the learning session. A closer look at the content analyses of the elaborative writing task reveals that even in EG 1, after the manipulation, many participants still associated errors with personal weakness that illustrates what one is not yet able to do or achieve, and many still emphasized the importance of avoiding errors (see the example provided from the written narratives). A more recent study by Pan et al. (2020) provides evidence for the ambivalent beliefs towards errors, showing that students (and teachers) believe that errors are useful for learning but at the same time tend to avoid errors and the deliberate generation of them. In general, it is questionable if long‐term effects can be achieved with such brief interventions. Future research should focus on longer term interventions (e.g., over the course of an academic year) and longitudinal study designs to capture students' attitude shifts in more detail. On the other hand, the mean scores for the belief that errors represent learning opportunities were generally quite high, even at baseline, and as well in the control group. Future research and interventions to promote positive beliefs about errors might particularly address learners who already show increased unfavourable attitudes in order to avoid ceiling effects.

As a consequence, we asked the question: Is it easier to directly influence adaptive responses to errors? In Study 2, we intertwined the manipulation more closely with the actual learning process by using prompts about adaptive responses immediately after giving error feedback. We found that those exposed to immediate messages about affective‐motivational adaptive responses to errors (exclusively or combined with prompts for adaptive action‐related responses to errors) differed significantly from the control group (and the action adaptivity prompt only‐group) in their persistence. This is in line with theory, such as the Dual Processing Model of Self‐Regulation (Boekaerts, 2006), because it shows that learners with maintained positive affect and motivation are more willing and ready to work on their errors. Our results further support previous findings, which highlight the negative impact of negative feelings and reduced motivation on student engagement and persistence (e.g., Eskreis‐Winkler & Fishbach, 2019). On the contrary, we provide additional evidence for the causal link between positive affect and motivation, and adaptive responses to errors. However, since the group differences for adaptive affective‐motivational reactions to errors after the manipulation were not significant, it remains questionable whether these reactions can be influenced by prompts.

As expected, prompts addressing adaptive action‐related responses to errors increased the level of metacognitive control and learners' intentions to take adaptive action in response to errors, but also their investment of effort. Thus, our findings contribute to the literature on prompts and self‐regulated learning (e.g., Bannert, 2009) as they underpin the importance of metacognitive and affective‐motivational support just‐in‐time.

We found no impact of our experimental manipulations on performance in a test of topic related knowledge. However, this is less surprising if we keep in mind that the effects that have been found in previous studies on error training (e.g., Keith & Frese, 2005) and on (metacognitive) prompts (e.g., Bannert et al., 2015) were often related to *transfer* performance. One limitation of our study lies in the type of performance test, focusing on recall and knowledge instead of using transfer tasks to operationalize performance and conceptual understanding. Although we found no effects on post‐test performance, the positive correlations between persistently and explicitly error‐centred learning behaviours and performance underline the relevance of action‐adaptive responses to errors for academic achievement. Future studies may develop more appropriate measurements of performance with the capacity to assess learning from errors (i.e., changes in conceptual understanding *in the longer term*) rather than recall of knowledge in the shorter term (see Kapur, 2016).

Finally, the distinction between learning and performance seems crucial: Research findings demonstrate that learners (and this may be true for teachers as well) tend to interpret performance during acquisition as an indicator of learning, which can lead to decreased motivation and misassessments of the degree to which (long‐term) learning has happened (Soderstrom & Bjork, 2015). Even more important would be the teachers' support of adaptive self‐regulation and learning behaviour and the maintenance of motivation following error feedback (Pan et al., 2020). It would therefore appear sensible to incorporate adaptive handling strategies for errors in learning activities and to provide scaffolding for learners when using them, particularly for adaptive action‐related responses to errors.

## CONCLUSION

Taken together, our results support and expand previous findings by providing a causal interpretation of the effects due to the experimental design. In doing so, new research questions emerged about how learners' adaptive responses to errors can best be supported, or how learners can best be prepared for error situations. Finally, Study 1 points particularly clearly to the unfavourable effects of negative beliefs about errors and argues for the benefits of raising awareness of this among students, teachers and parents who might hold unfavourable convictions such as “errors disturb the learning process, draw attention to inaccurate facts and cause learners to retain these preferentially”. The results of Study 2 emphasize the importance of supporting students in developing adaptive affective‐motivational reactions and adaptive learning behaviour immediately following errors.

## AUTHOR CONTRIBUTIONS

**Maria Tulis:** Writing – original draft; conceptualization; investigation; methodology; writing – review and editing; project administration; formal analysis; data curation; visualization; funding acquisition. **Markus Dresel:** Conceptualization; writing – review and editing; supervision.

## CONFLICT OF INTEREST STATEMENT

All authors declare that they have no conflicts of interest.

## Supporting information


**Data S1.** Supporting information.

## Data Availability

The data that support the findings of this study are available from the corresponding author upon reasonable request.
