# Supplementary material for: Effects on and consequences of responses to errors: Results from two experimental studies
Source: Br J Educ Psychol. 2024 May 8;95(1):143–61. doi: 10.1111/bjep.12686 (PMC11802961; doi:10.1111/bjep.12686)
Supplement: Supplementary file 1 — Data S1. Supporting information. [file BJEP-95-143-s001.docx]

**Supplemental material**

**S1**. Knowledge pretest

**S2**. Example booklet “tips for learning” (“positive error-related beliefs”-manipulation in Study 1)

**S3**. Examples of manipulation step 2 (typical student figures modelling error-related beliefs) for EG 1 (Figure 1a) and EG 2 (Figure 1b) in Study 1

**S4**. Examples of the post-it notes in Study 1

**S1. Knowledge pretest** (translated in English by the first author)

1. *Evaluate the accuracy of the following statements:*

A) The correlation coefficient r ranges between 1 and -1.

B) Hypothetical constructs are variables that cannot be observed directly.

C) Hypothetical constructs are theories that cannot be confirmed.

D) If *p* ≥ .05, an effect is "non-significant" and the hypothesis in question is rejected.

*2. Which of the following statements about research designs is/are correct?*

1. Randomisation is the random assignment of test subjects to experimental conditions; it is a central feature of the experimental method.
2. A quasi-experiment is an experiment without randomisation.
3. Randomisation is a method for recording the dependent variable.
4. An experiment is used to test the extent to which the manipulation of the independent variable causes changes in the dependent variable.

*3. A cross-sectional study of students revealed a significant correlation of r = .63 between interest in the subject of psychology and performance in a psychology exam. What conclusion(s) can be drawn directly from this finding?*

1. Students who are interested in psychology often tend to perform well in this subject.
2. A clear interest in psychology results in high performance in psychology.
3. Poor performance in psychology causes a decline in interest in psychology.
4. There is no significant correlation between interest in psychology and performance in psychology.

*4. Please assess the correctness of the following statements:*

1. Objectivity describes the extent to which the result of a study is not influenced by the person conducting the study.
2. Inferential statistics allow conclusions to be drawn from the relationships in the analysed sample to the population of interest.
3. Reliability refers to the degree to which a measurement produces consistent results.
4. The smaller the sample, the smaller the sampling error.

**S2. Example booklet “tips for learning” from EG 1, Study 1** (in German language)

<http://online.fliphtml5.com/zlibl/tqpj/#p=12>


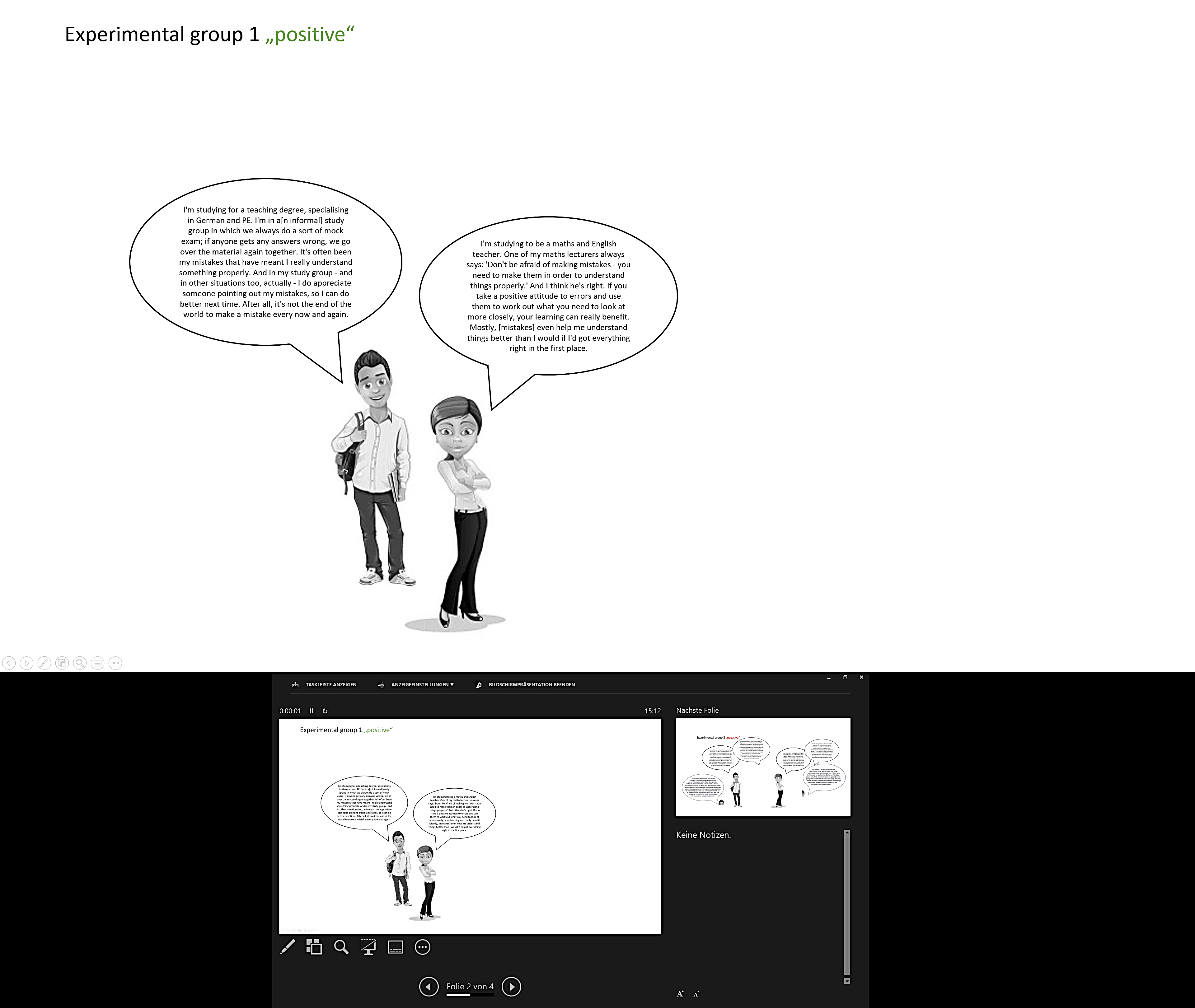


**S3. Figure 1a.** Examples of manipulation step 2 (typical student figures modelling error-related beliefs) for EG 1 (positive beliefs) in Study 1.


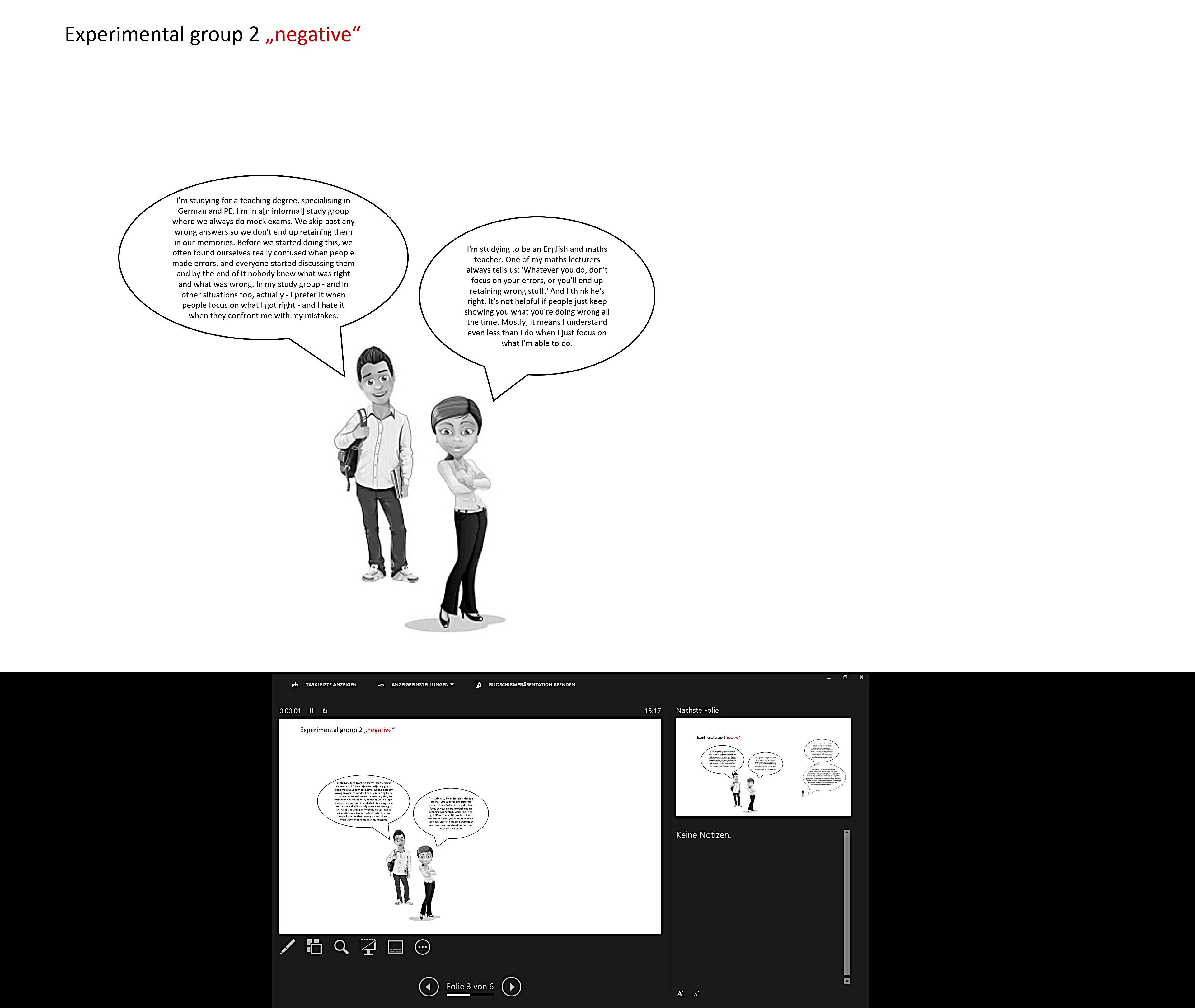


**S3. Figure 1b.** Examples of manipulation step 2 (typical student figures modelling error-related beliefs) for EG 2 (negative beliefs) in Study 1.


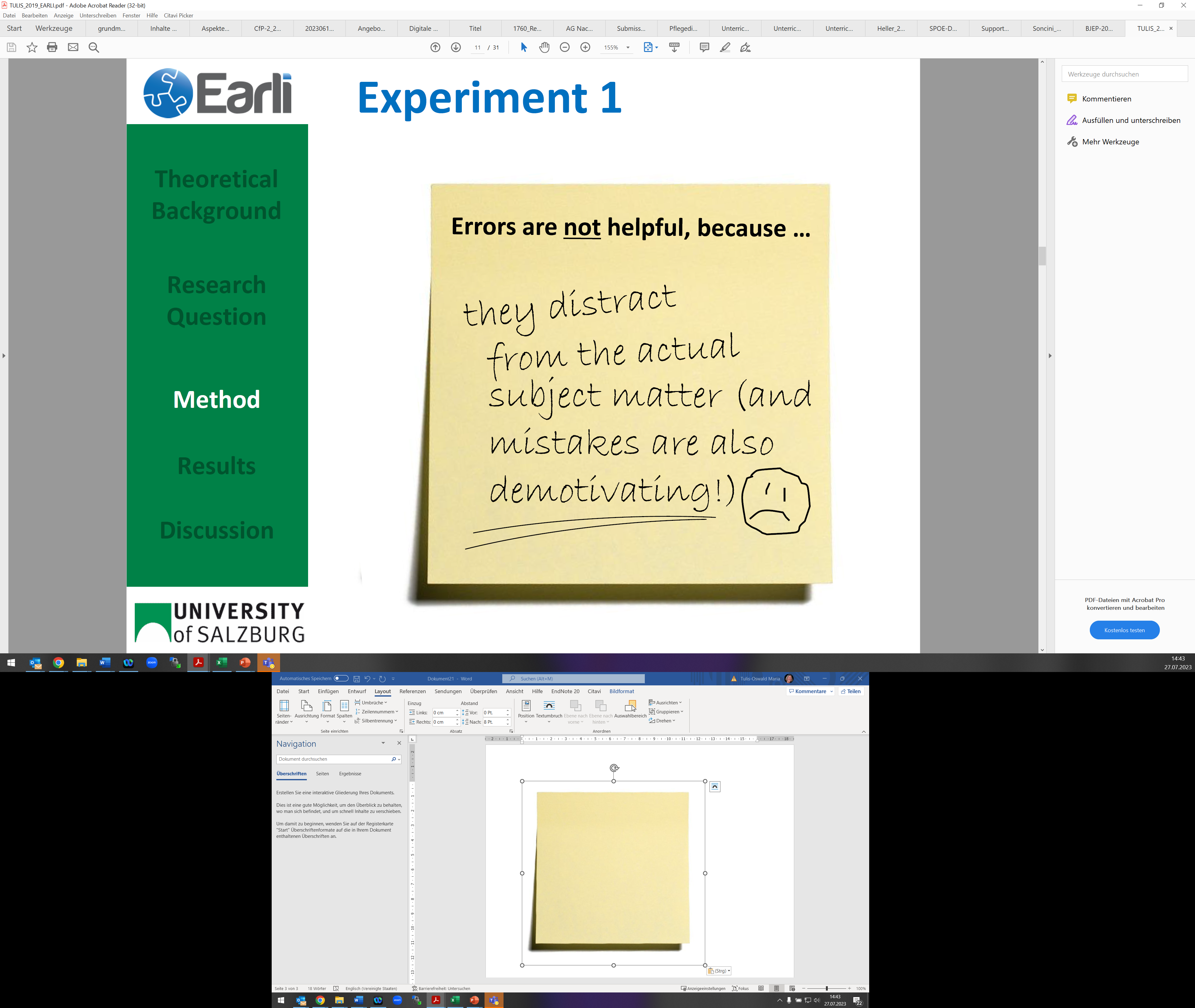

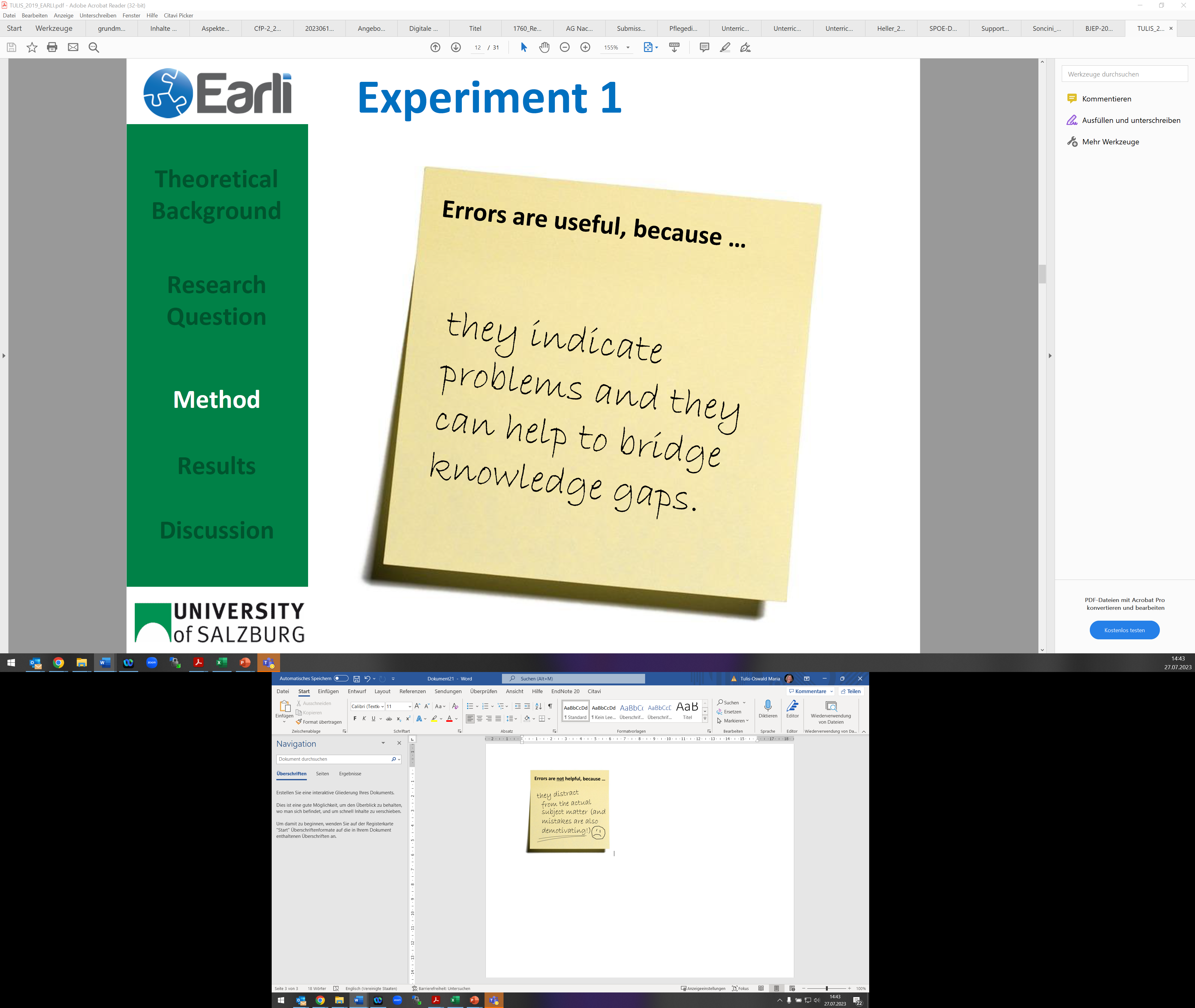


**S4. Figure 2.** Examples of the post-it notes in Study 1.

*Note.* Written by a participant of EG 2 (on the left) and a participant of EG 1 (on the right).
